# Empathy and Autism: Establishing the Structure and Different Manifestations of Empathy in Autistic Individuals Using the Perth Empathy Scale

**DOI:** 10.1007/s10803-024-06491-3

**Published:** 2024-08-08

**Authors:** Jack D. Brett, David A. Preece, Rodrigo Becerra, Andrew Whitehouse, Murray T. Maybery

**Affiliations:** 1 The University of Western Australia, Perth, Australia; 2 School of Psychology, Curtin University, Perth, Australia; 3 Telethon Kids Institute, The University of Western Australia, Perth, Australia; 4 Cooperative Research Centre for Living with Autism (Autism CRC), Perth, Australia

**Keywords:** Autism spectrum disorders, Empathy, Moderated non-linear factor analysis, Adults, Measurement, Social cognition and social behavior

## Abstract

**Purpose:**

There is a common mischaracterisation that autistic individuals have reduced or absent empathy. Measurement issues may have influenced existing findings on the relationships between autism and empathy, and the structure of the empathy construct in autism remains unclear.

**Methods:**

The present study sought to address these gaps by examining the structure and psychometric properties of the Perth Empathy Scale (PES) in autistic individuals (*N* = 239) compared to non-autistic individuals (*N* = 690).

**Results:**

Our moderated non-linear factor analysis revealed that the multidimensional empathy construct manifested similarly in autistic and non-autistic individuals, with the PES displaying good validity and reliability. Moreover, the results revealed that autistic individuals reported reduced cognitive empathy and reduced affective empathy for positive and negative emotions. However, there was greater heterogeneity of empathic tendencies in the autistic sample, indicating that these mean differences may not be generalisable for all autistic individuals.

**Conclusion:**

The present study highlights that the PES is suitable for assessing empathy across autistic and non-autistic individuals. This work with the PES also provides greater nuance to our understanding of empathy and autism, and based on these findings, we propose the *empathy heterogeneity hypothesis of autism* as a new way of describing empathy in autism.

A pervasive stereotype is that autistic individuals^1^ have reduced or absent experiences of empathy (Fletcher-Watson & Bird, 2020). Part of this misconception may stem from the fact that while it is common to investigate empathic differences with self-report questionnaires (Fatima & Babu, 2023), there has not yet been a measure of empathy directly validated for use in autistic samples (Harrison et al., 2022). Thus, the accuracy and understanding of empathy measurement in this population is uncertain. The current paper aims to provide a potential remedy for this by validating the Perth Empathy Scale (Brett et al., 2023), enabling it to be used to establish the structure of the multidimensional empathy construct and examine empathy profiles across autistic and non-autistic individuals.

Recent reviews of empathy (e.g., Cuff et al., 2016; Eklund & Meranius, 2021) highlight the lack of a universally agreed definition. Nonetheless, taking together and critically appraising definitions of empathy, Cuff et al. (2016) provide some consensus that empathy consists of cognitive and affective components. Cognitive empathy is the ability to infer and recognise another’s affective state (e.g., Brett et al., 2023; Coll et al., 2017; Ickes, 1993; Innamorati et al., 2019; Vachon & Lynam, 2016) and affective empathy is the ability to share in another’s affect while being aware that the emotional experience is in response to another’s (e.g., Brett et al., 2023; de Vignemont & Singer, 2006). In this context, Smith (2009) proposed the *empathy imbalance hypothesis of autism*, which posits that autistic individuals might have difficulty in cognitive empathy but show intact or even heightened affective empathy. However, a recent meta-analysis (Song et al., 2019) indicated that autistic individuals report more difficulties than non-autistic individuals in both affective and cognitive empathy on self-report measures.

To add further nuance, empathising with others’ positive or negative emotions appears to reflect distinct capabilities (Andreychik & Migliaccio, 2015) and arise from separable neurological processes (Morelli et al., 2014; Ziaei et al., 2021). For example, using the Multifaceted Empathy Test (Dziobek et al., 2008), Quinde-Zlibut et al., (2021) showed that autistic individuals struggled to infer (i.e., cognitively appraise) others’ emotions, irrespective of valence, but showed increased affective empathy for negative emotions and reduced affective empathy for positive emotions, compared to non-autistic individuals, although this result is not always consistent and many empathy measures used in the field do not consider both negative and positive valences (see Mazza et al., 2014).

Overall, the findings on the empathy profile of autistic individuals are therefore somewhat inconsistent but tend to reflect more difficulties with cognitive empathy across both valence domains, affective empathy for positive emotions, and potentially enhanced affective empathy for negative emotions compared to non-autistic individuals (Fatima & Babu, 2023; Quinde-Zlibut et al., 2021; Song et al., 2019). However, before the empathy profile of autism can be confidently explored, it must first be established that there are psychometric tools that can validly and reliably assess empathy in autistic individuals.

Empathy is often measured using self-report questionnaires, and 91% of the studies identified in a recent review (Fatima & Babu, 2023) used questionnaire-based measures to assess differences in cognitive and affective empathy between autistic and non-autistic individuals. However, no previous empathy measure has shown adequate psychometric properties to assess empathy in autistic individuals (Harrison et al., 2022) nor measurement invariance between autistic and non-autistic groups (Fatima & Babu, 2023). Harrison et al. (2022) argued that many empathy measures use nonliteral language (e.g., “touched by things”) and imprecise language, which may bias responses for autistic individuals given potential difficulties interpreting this language (Gold et al., 2010; Olofson et al., 2014). However, a new empathy self-report measure, the Perth Empathy Scale (PES; Brett et al., 2023), has recently been validated within the general population.

The PES is a 20-item self-report measure that assesses if one can accurately recognise others’ emotions (cognitive empathy) and whether an emotion in someone else creates that emotion in oneself (affective empathy). The PES avoids nonliteral language by directly asking respondents about their experiences empathising with specific emotions. The cognitive empathy items are worded as “Just by seeing or hearing someone, I know if they are feeling [an emotion]”, while the affective empathy items are worded as “When I see or hear someone who is [an emotion], it makes me feel [the same emotion] too”. Five negatively valenced emotions (i.e., sad, angry, scared, disgusted, and embarrassed) and five positively valenced emotions (e.g., happy, amused, calm, enthusiastic, and pride) are used.

The PES has shown good psychometric properties from its initial development (Brett et al., 2023) and in other languages (e.g., Larionow & Preece, 2023) within the general population. These previous psychometric studies of the PES showed that individuals did not discriminate between positive and negative emotions for cognitive empathy but did distinguish between positive and negative emotions for affective empathy. Overall, factor analyses suggested that empathy consists of a total empathy factor together with a general cognitive empathy factor and valence-specific affective empathy factors.

## Comprehensive Assessment of the Empathy Construct

One valuable and contemporary methodology for examining the structure of empathy and its manifestations across autistic and non-autistic individuals is Moderated Non-Linear Factor Analysis (MNLFA; Bauer, 2017). MNLFA assesses measurement invariance (or differential item functioning; see Bauer, 2017) and latent construct differences between groups and as a function of continuous variables. MNLFA allows a comprehensive investigation of latent construct differences by assessing how factors (categorical or continuous) influence construct means (i.e., if a latent construct is more common in one group compared to another), variances (i.e., if a latent construct varies more in one group over another), and covariances (i.e., if two latent constructs are more distinct in one group compared to another). Researchers often investigate mean differences, as is the case for the previous research conducted between autistic and non-autistic individuals (e.g., Fatima & Babu, 2023; Song et al., 2019), but due to limitations in previous statistical analyses (now resolved by the introduction of MNLFA) existing studies had been unable to assess differences in variances and covariances. This is a significant limitation, particularly when involving highly heterogeneous groups such as the autistic population.

Researchers, clinicians, and autistic individuals often note the importance of considering the significant differences between autistic individuals (e.g., Georgiades et al., 2013; Hassan & Mokhtar, 2019; Masi et al., 2017; Mottron & Bzdok, 2020; Tillmann et al., 2020). While there have been attempts to identify autistic subgroups (e.g., Georgiades et al., 2013), few studies have investigated whether the autistic population shows greater variability in characteristics compared to the non-autistic population. Investigating whether there is greater heterogeneity in empathic tendencies in the autistic population than in the non-autistic population provides a nuanced understanding of how empathy is represented within the autistic population. For instance, while an autistic sample may report reduced empathic tendencies, heterogeneity could imply that this mean difference may not adequately characterise the autistic population. Indeed, evidence of greater heterogeneity may suggest additional characteristics that moderate empathic differences.

Lastly, MNLFA can assess covariance differences, that is, whether latent constructs relate differently depending on individuals’ characteristics. MNLFA allows an examination of whether constructs are more (or less) distinct in the autistic population than in the non-autistic population. Regarding empathy, it is unknown whether autistic individuals show more distinction between cognitive and affective empathy, whereby autistic individuals with reduced cognitive empathy may not necessarily show reduced affective empathy (compared to non-autistic individuals). Additionally, it is possible that autistic individuals could show less valence-specificity in affective empathy (i.e., reporting more similar experiences of affective empathy regardless of the valence). These exciting questions were not easily assessed until the development of MNLFA, and thus this methodology has several significant advantages that can be harnessed to advance the field.

## Moderating Effects of Age and Gender

In addition to comprehensively assessing the empathy profile of autism, MNLFA can also be used to examine the moderating effects of additional characteristics, such as age and gender. In a meta-analysis investigating empathic differences between autistic and non-autistic individuals, Song et al. (2019) noted that age and gender moderated empathic differences. Reported age effects for empathy in non-autistic and autistic populations have been variable. In non-autistic individuals, some studies suggest that affective and cognitive empathy increase with age (Khanjani et al., 2015; Oh et al., 2020; Tremblay et al., 2022), although others suggest there is no change or reduced empathy with age (Bailey et al., 2020; Pollerhoff et al., 2022). While there may not be a consistent age effect, Song et al. (2019) indicated that autistic and non-autistic individuals may not show the same age trends in empathy. Specifically, they suggested that autistic individuals report greater increases in affective empathy with age compared to non-autistic individuals; however, they report a greater decrease in cognitive empathy with age than non-autistic individuals.

Regarding gender differences in empathy, Song et al. (2019) suggest that gender moderates the difference between autistic and non-autistic individuals on affective empathy. While autistic individuals may report the same or more affective empathy than non-autistic individuals (Song et al., 2019), autistic females report significantly more affective empathy than expected, given the non-autistic gender difference. This finding is in line with a growing body of research investigating the female autism phenotype (see Hull et al., 2020), which has highlighted that autistic females show differences in their manifestation of autism, such as greater social motivation (Hiller et al., 2014), greater interest in relations (Lawson, 2019), greater internalising problems (Chandler et al., 2016), less externalising problems (Hiller et al., 2014), and greater motivation to camouflage (Hull et al., 2020) than autistic males. Indeed, some preliminary findings have indicated that autistic trait-related empathic differences may be underpinned by different mechanisms for females and males (Brett et al., 2024).

## The Present Study

The present study aims to provide comprehensive insight into the structure and profile of empathy in autistic individuals. In doing so we investigate multiple hypotheses regarding how empathy (as measured by the PES) manifests in the autistic population, whether the PES biases scores for autistic individuals, whether there are mean, variance, or covariance differences between autistic and non-autistic individuals, and whether these differences are moderated by age or gender.

Firstly, it was hypothesised that empathy manifests similarly in autistic individuals as it does in non-autistic individuals. As such, the factor structure of PES in autistic individuals was predicted to show a general cognitive empathy factor (across both negative and positive emotions) and two valence-specific affective empathy factors split across negative and positive emotions (as suggested by Brett et al., 2023). Additionally, through bifactor analyses, the presence of an overarching empathy factor would be tenable. Secondly, it was hypothesised that the PES is a valid assessment of empathy, which does not unduly bias scores for autistic individuals. As such, it was predicted that the PES would exhibit limited differential item functioning due to autistic diagnosis status, including when investigating the interacting effects of gender and age (e.g., bias for only autistic females).

Thirdly, it was hypothesised that mean empathy differences would align with Quinde-Zlibut et al. (2021). As such, it was predicted that autistic individuals, on average, would report a reduced tendency for cognitive empathy, a reduced tendency for affective empathy for positive emotions, but an increased tendency for affective empathy for negative emotions. Fourthly, it was hypothesised that empathy heterogeneity would be present in the autistic population. As such, we predicted that the autistic population would exhibit greater variability in their empathic tendencies, in line with the high heterogeneity often observed within this population for other constructs (e.g., Georgiades et al., 2013; Masi et al., 2017; Mottron & Bzdok, 2020; Hassan & Mokhtar, 2019; Tillmann et al., 2020). Fifthly, it was hypothesised that the empathy factors relate similarly across autistic and non-autistic populations. As such, we predicted that the relationships between empathy factors would not vary as a function of autism diagnostic status.

Lastly, it was hypothesised that age and gender would moderate the relationships between autism diagnosis status and empathy. In line with Song et al.’s (2019) findings, it was predicted that the reported age-related decreases in cognitive empathy would be greater in the autistic population than in the non-autistic population. It was also predicted that the reported age-related increases in affective empathy would be greater in the autistic population than in the non-autistic population. Regarding gender, it was predicted that autistic females would report greater affective empathy than autistic males, and to a greater extent than is observed between non-autistic females and males.

## Method

### Participants

All participants^2^ completed the Perth Empathy Scale (PES) online through Qualtrics software. An autistic sample consisting of 239 individuals having an independent clinical diagnosis of autism (from a psychiatrist, psychologist, or other qualified medical specialist). A non-autistic sample of 690 participants was taken from the original Brett et al. (2023) PES validation studies. Individuals who reported self-identifying as autistic without a formal diagnosis were excluded from this non-autistic sample. Unfortunately, gender-diverse individuals (e.g., non-binary or genderfluid) also needed to be excluded from the analyses to meet statistical assumptions due to inadequate sample size to assess this group of people (i.e., 2.4% of the total sample).

Demographic information for these two samples is provided in Table 1. Differences between groups were examined through regression analyses (Table 1), which showed that the groups differed in age (the autistic group being older) and the proportion of females (the autistic group having fewer females). By including age and gender in the MNLFA, these effects are statistically controlled for, although a sensitivity analysis was also completed using a matched sample (see Supplementary Material). Overall, both samples came predominantly from Western countries (e.g., United States of America, Britain, Europe, and Australia) without significant diversity (i.e., only 0.93% were Australian First Nation Peoples). Participants provided informed consent digitally, and the study was designed according to the principles of the Declaration of Helsinki.

**Table 1:** Demographic variables for the autistic and non-autistic samples

	Autistic sample	Non-autistic sample	Comparison
Age in years (SD)	32.89 (10.39)	26.01 (10.82)	B = 6.88, *p* < .001
% Female	45.6	69.4	B = -1.00, *p* < .001

*Note* SD = Standard deviation; B = unstandardised beta weight. Comparisons were investigated using logistic regressions where age or gender predicted autism diagnosis. Significant beta-weights suggest significant differences between groups on these variables

### Materials

The Perth Empathy Scale (PES; Brett et al., 2023) is a 20-item self-report questionnaire that assesses empathy across cognitive and affective components and does so for both negative and positive emotional valences. The items are paired for ten specific emotions (i.e., sad, happy, angry, amused, scared, calm, disgusted, enthusiastic, embarrassed, & proud) with one item each asking about each emotion for cognitive empathy (e.g., “Just by seeing or hearing someone, I know if they are feeling sad”) or affective empathy (e.g., “When I see or hear someone who is sad, it makes me feel sad”). In the general population (Brett et al., 2023; see also Larionow & Preece, 2023), the factor structure of the PES shows a general cognitive empathy factor and valence-specific affective empathy factors, and that a general empathy factor is tenable. The PES uses a 5-point Likert scale, with higher scores indicating greater empathy.

### Statistical Analyses

**Fig. 1:**
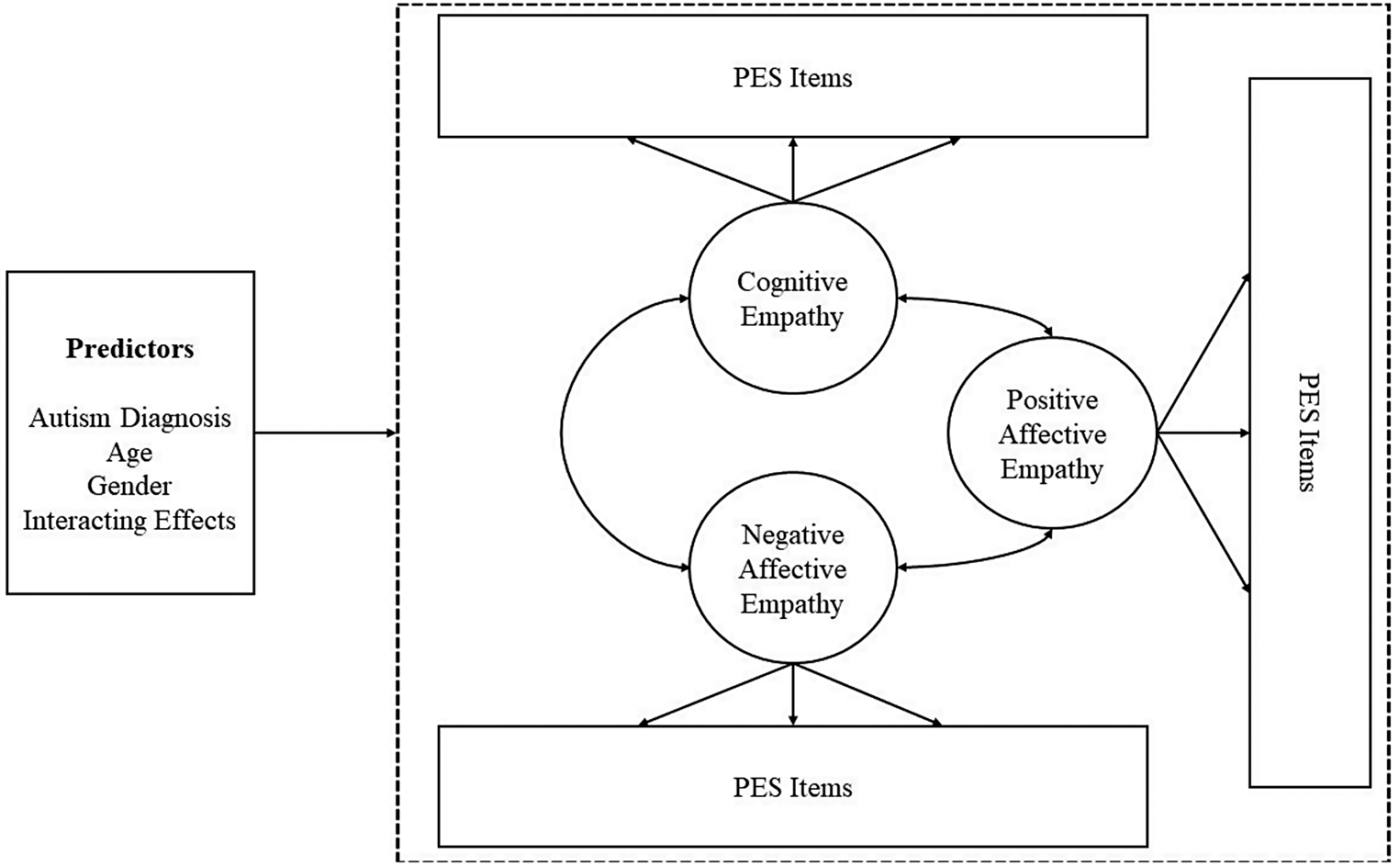
Conceptual diagram of the multi-dimensional MNLFA model. *Note* Factor variances and item residual variances are not modelled. The arrow pointing to the dashed rectangle conveys that the values for all model parameters (except for item residual variances) were specified as a function of the predictors and their two-way interactions

The present study investigated the PES using Confirmatory Factor Analyses (CFAs) and Moderated Non-Linear Factor Analysis (MNLFA). The CFA and MNLFA analyses were completed through Mplus (version 8.0). Items were treated as ordinal categorical variables (Rhemtulla et al., 2012). Figure 1 provides an illustration of the MNLFA conducted. To conduct this analysis, we used the steps suggested by Bauer (2017; we recommend that interested readers refer to this for more detail). Age was centred around the grand mean (i.e., 27.78 years). A log-linear transformation was applied to the variances, and Fisher’s z transformation was applied to the covariances. The sample size in the present study provided sufficient power as determined by published peer-review simulation papers investigating a variety of factors influencing power in MNLFA and related analyses (*N* = 200–500; e.g., Bauer et al., 2020; Gottfredson et al., 2019; Kolbe et al., 2021). R studio (version 4.3.0) was used to conduct the Johnson-Neyman regions of significance analyses using the package *interactions* (version 1.1.5), and data visualisation was completed using the package *ggplot2* (version 3.3.0). SPSS 29 was used to compute internal consistency reliabilities.

### Hypothesis 1: Factor Structure

First, we assessed the factor structure of the PES within the autistic sample using confirmatory factor analyses. We examined different theoretically driven models: (1) a unidimensional one-factor model with a single “general empathy” factor, (2) a two-factor model of “affective empathy” and “cognitive empathy”, (3) a four-factor model of valence-specific affective and cognitive empathy (“negative cognitive empathy”, “positive cognitive empathy”, “negative affective empathy”, “positive affective empathy”), (4) the three-factor model supported in Brett et al. (2023), comprised of a “general cognitive empathy” factor and valence-specific “negative affective empathy” and “positive affective empathy” factors, and (5) a bifactor variant of the best fitting model to enable a “general empathy” factor to be represented alongside narrower subscale factors. The models were constructed to account for emotion-specific empathy (as suggested by Olderbak et al., 2014; i.e., allowing items measuring the same emotion to covary given their similarities in item content). These configural models were estimated using the weighted least square mean and variance adjusted (WLSMV) estimator to obtain model fit indices.

To determine the appropriateness of interpreting a total score of the PES for general empathy, we calculated omega hierarchical (*ω*_*H*_) to obtain the proportion of variance in the total score accounted by general empathy and explained common variance of the general factor (ECV_G_), using the Dueber (2017) bifactor indices calculator. *ω*_*H*_ values *≥* 0.70 and ECV_G_*≥* 0.60 provide strong evidence of a general factor (Reise et al., 2013; Revelle & Wilt, 2013). Internal consistency reliabilities were also obtained using total omega and alpha coefficients.

### Hypothesis 2: PES Bias

A multi-dimensional MNLFA (Fig. 1) was then developed to assess DIF using the robust categorical maximum likelihood estimator (cat-MLR) that Bauer (2017) suggested. To reduce computational demands, unidimensional MNLFAs were run for each empathy factor to provide starting values for a multi-dimensional MNLFA (these unidimensional MNLFAs can be found in Appendix A). Item DIF was evaluated in an iterative (stepwise procedure) whereby one item was examined simultaneously with all other items anchored to be DIF-free. All predictors (autistic diagnosis status, gender, and age) and their interactions were considered simultaneously. Improvements in model fit based on likelihood ratio tests (LRTs; Satorra & Bentler, 2001), with a Benjamini-Hochberg procedure to control for the false discovery rate (Thissen et al., 2002), indicated that an item exhibited statistically significant DIF. After identifying items exhibiting DIF, we removed non-significant DIF terms, except for lower-order terms, if a significant interaction was found (based on LRTs). This procedure of identifying DIF provides conservative results, thus identifying items that may not provide significant DIF (Bauer et al., 2021) but allows greater confidence in the PES measurement.

There is no consensus on what constitutes partial invariance for MNLFA. However, to align with the COSMIN guideline for measures (Prinsen et al., 2018), we computed McFadden’s R^2^ to indicate the magnitude of the DIF. Prinsen et al. (2018) recommend that important DIF occurs once McFadden’s R^2^ exceeds 0.02; if below this threshold, we report the DIF as non-meaningful.

### Hypotheses 3–6: Empathy Profile of Autism and Moderating Effects

While the multidimensional MNLFA was used to examine DIF, it also enabled an investigation into the empathy profile of autism by highlighting what predictors (e.g., autism, age, and gender) and their interactions influence the empathy factors’ means, variances, and covariances. As Bauer (2017) suggested, all predictors (and two-way interactions) were initially allowed to influence factors’ means, variances, and covariances, and we removed non-significant effects in a backward stepwise procedure (based on LRTs). A model was preferred if it did not show a significant difference compared to the nested model (i.e., the more parsimonious model was preferred if there was no difference in model fit). Hedges’ g was computed as the effect size for the mean differences (Brydges, 2019). Additionally, the moderating impacts of age and gender were investigated (e.g., whether age moderated differences between autistic and non-autistic individuals in empathy). When a significant interaction effect was found, simple main effect analyses were conducted whereby the model was re-run, isolating the groups of interest. Additionally, Johnson-Neyman regions of significance were conducted on interactions with continuous variables (i.e., age).

## Results

### Hypothesis 1: Factor Structure

Multiple confirmatory factor analyses were conducted to assess configural invariance. The model that best fit the autistic sample was the 4-factor model (see Table 2). However, the correlation between negative and positive cognitive empathy was 0.97, suggesting that the factors measured the same construct. As such, the 3-factor model, identified by Brett et al. (2023), was judged the most appropriate. The bifactor variant of this 3-factor solution produced adequate fit, suggesting that including a general empathy factor was tenable (See Supplementary Material Table S2 for factor loadings). The bifactor model provided *ω*_*H*_ = 0.73 and ECV_G_ = 0.569. These values indicate that while the general empathy factor accounts for a large amount of systematic variance (i.e., *ω*_*H*_), a proportion of the common variance explained by the subscales would be missed if only using the general empathy factor. As such, the parsimonious first-order factor model was used in the MNLFA due to significant computational demands for an ordinal bifactor MNLFA and based on the bifactor model indices. Nonetheless, the subscales showed acceptable to excellent internal consistency reliability for autistic individuals (Table 3).

**Table 2:** Model fit indices for the alternative empathy models in the autistic sample

Model	df	χ^2^	RMSEA [90% CI]	CFI	TLI
Unidimensional	160	1093.51	0.156 [0.148, 0.165]	0.880	0.858
2-factor	159	397.85	0.079 [0.070. 0.089]	0.969	0.963
3-factor	157	357.62	0.073 [0.063, 0.083]	0.974	0.969
4-factor*	154	331.87	0.070 [0.059, 0.080]	0.977	0.972
3-factor bifactor	139	318.97	0.074 [0.063, 0.084]	0.977	0.968

*Note* df = degrees of freedom; RMSEA = Root-Mean Square Error Approximation; CFI = Confirmatory Fit Index; TLI = Tucker-Lewis Index

*Negative and positive cognitive empathy correlated at 0.97

**Table 3:** Raw score means, standard deviations, and internal consistency reliabilities of the PES subscales

Scale	Mean (SD)			Omega (alpha)	
	Autistic	Non-autistic		Autistic	Non-autistic
CE	27.78 (9.74)	37.90 (7.21)		0.95 (0.95)	0.92 (0.92)
NAE	10.94 (4.47)	12.16 (3.75)		0.82 (0.81)	0.75 (0.75)
PAE	11.98 (4.88)	15.87 (4.01		0.86 (0.86)	0.78 (0.78)
Empathy	50.70 (16.25)	65.93 (11.47)		0.94 (0.94)	0.88 (0.89)

*Note* CE = Cognitive Empathy; NAE = Negative Affective Empathy; PAE = Positive Affective Empathy. Omega hierarchical for the total empathy score was 0.73 for the autistic sample and 0.54 for the non-autistic sample

### Hypothesis 2: PES Bias

The multi-dimensional MNLFA highlighted that the structure of the PES showed non-meaningful (i.e., McFadden’s R^2^ < 0.02; Prinsen et al., 2018) partial invariance between autistic and non-autistic individuals (see Supplementary Materials). As such, having an autism diagnosis did not meaningfully bias scores on the PES, including after accounting for the potential interacting effects of gender and age (e.g., no meaningful bias for only autistic females). This result provides greater confidence that scores on the PES are due to underlying empathic tendencies (i.e., measuring empathy in the same way across groups).

### Empathy Profile of Autism

**Fig. 2:**
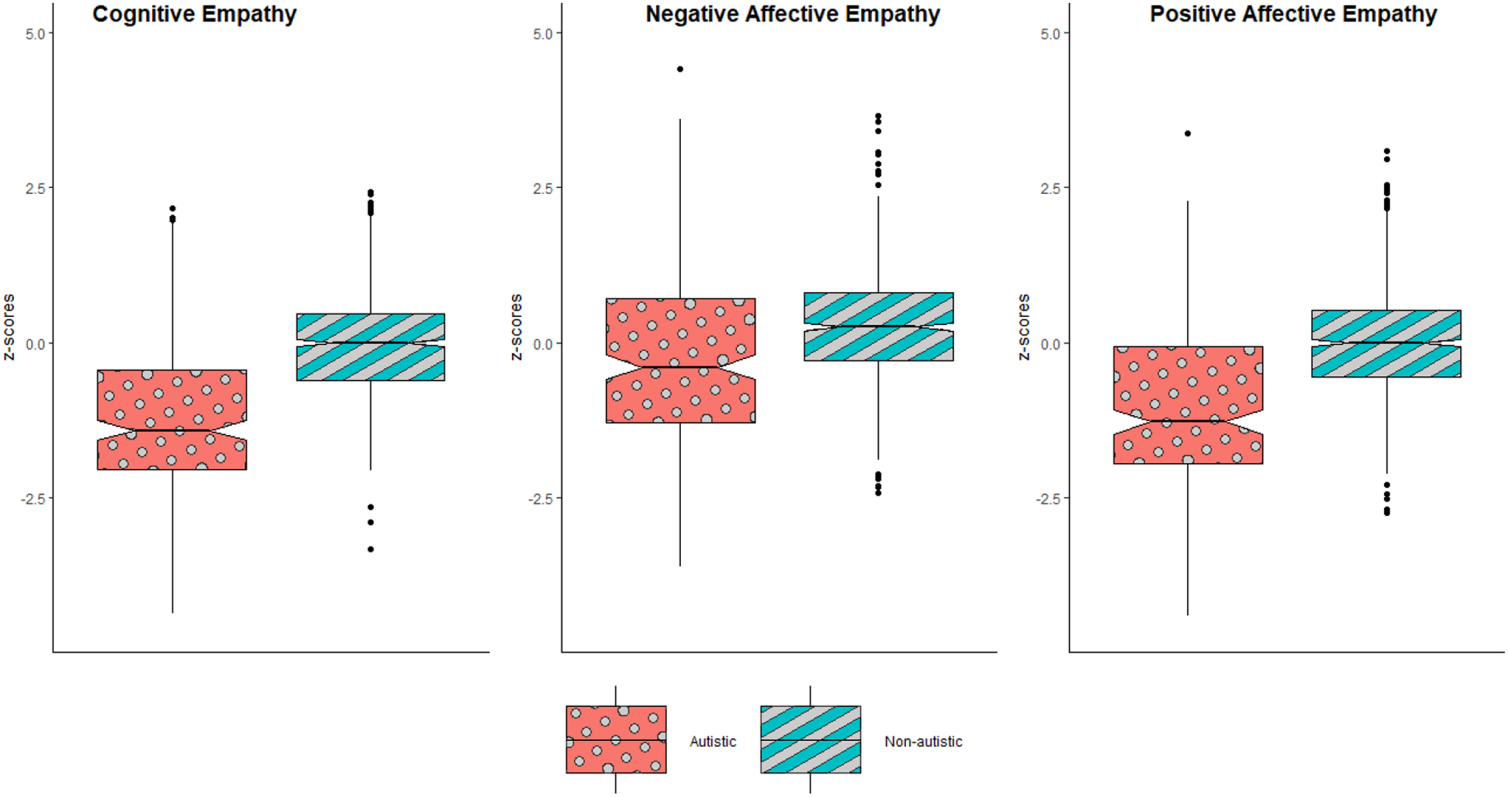
Multidimensional MNLFA factor scores across autistic and non-autistic individuals for cognitive and valence-specific empathy. *Note* Outliers (i.e., 1.5 times the interquartile range) are displayed with dots

While autism diagnosis did not meaningfully bias scores on the PES, the MNLFA continues to control for the potential influence of DIF, gender, and age. Table 4 provides the structural parameters, and Fig. 2 shows the MNLFA factor scores comparing the autistic and non-autistic groups.

**Table 4:** Influence of study variables on the empathy factors’ means and variances

Reference Parameter	Baseline	Covariate effect			
		Autism	Age	Gender	Age x Autism
Cognitive Empathy					
Mean	0.00^a^	-1.13***	0.01*	-	-0.03***
Variance	1.00^a^	1.36	-	-	-
Negative Affective Empathy					
Mean	0.00^a^	-0.59***	-0.02***	0.35***	0.03***
Variance	1.00^a^	2.21***	-	-	-
Positive Affective Empathy					
Mean	0.00^a^	-1.15***	-	-	-
Variance	1.00^a^	2.00***	-	-	-

*Note* The interacting effects of “Autism x Gender” and “Age x Gender” were investigated but showed no effect on the parameters and are not included in the table. The baseline indicates where all study variables are 0 (age was centred around the mean of 27)

### Hypothesis 3: Influence of Autism Diagnosis on Means

After controlling for gender and age, there was a large difference in cognitive empathy means, with autistic individuals reporting lower cognitive empathy than non-autistic individuals (Hedges g = -1.08). There was also a large difference in means for affective empathy for positive emotions, with autistic individuals reporting a reduced tendency (Hedges g = -1.03). Lastly, there was a medium-sized group difference in affective empathy for negative emotions, with autistic individuals reporting a reduced tendency here as well (Hedges g = -0.51).

### Hypothesis 4: Influence of Autism Diagnosis on Variance

There was greater variability in the autistic sample for negative and positive affective empathy than in the non-autistic sample. However, autism diagnosis did not show a statistically significant effect on the variance for cognitive empathy (Table 4). Thus, overall, there is significant heterogeneity in affective empathy in autistic compared to non-autistic individuals. As such, the overall effect of autism on valence-specific affective empathy should be taken with some caution, as these abilities are significantly more varied in the autistic population (e.g., some autistic individuals may show exceptional affective empathy, while others may show more difficulties) and likely depend on additional constructs (i.e., moderating effects).

### Hypothesis 5: Influence of Autism Diagnosis on Factor Correlations

The correlations between cognitive empathy and negative affective empathy (*r* = .35, *p* < .001) and between cognitive empathy and positive affective empathy (*r* = .48, *p* < .001) were equivalent between autistic and non-autistic individuals (i.e., model fit did not worsen when predictors were forced not to influence these covariances)^3^. However, individuals on the spectrum showed a larger positive correlation between negative and positive affective empathy than non-autistic individuals (*r*_*autistic*_ = 0.81, *p* < .001; *r*_*non−autistic*_ = 0.61, *p* < .001; *r*_*Δ*_ = 0.20, *p* < .003). As such, while there continues to be valence-specific affective empathy, autistic individuals report less distinction between these two valences for affective empathy than non-autistic individuals.

### Hypothesis 6: Moderating Effects of Age and Gender

**Fig. 3:**
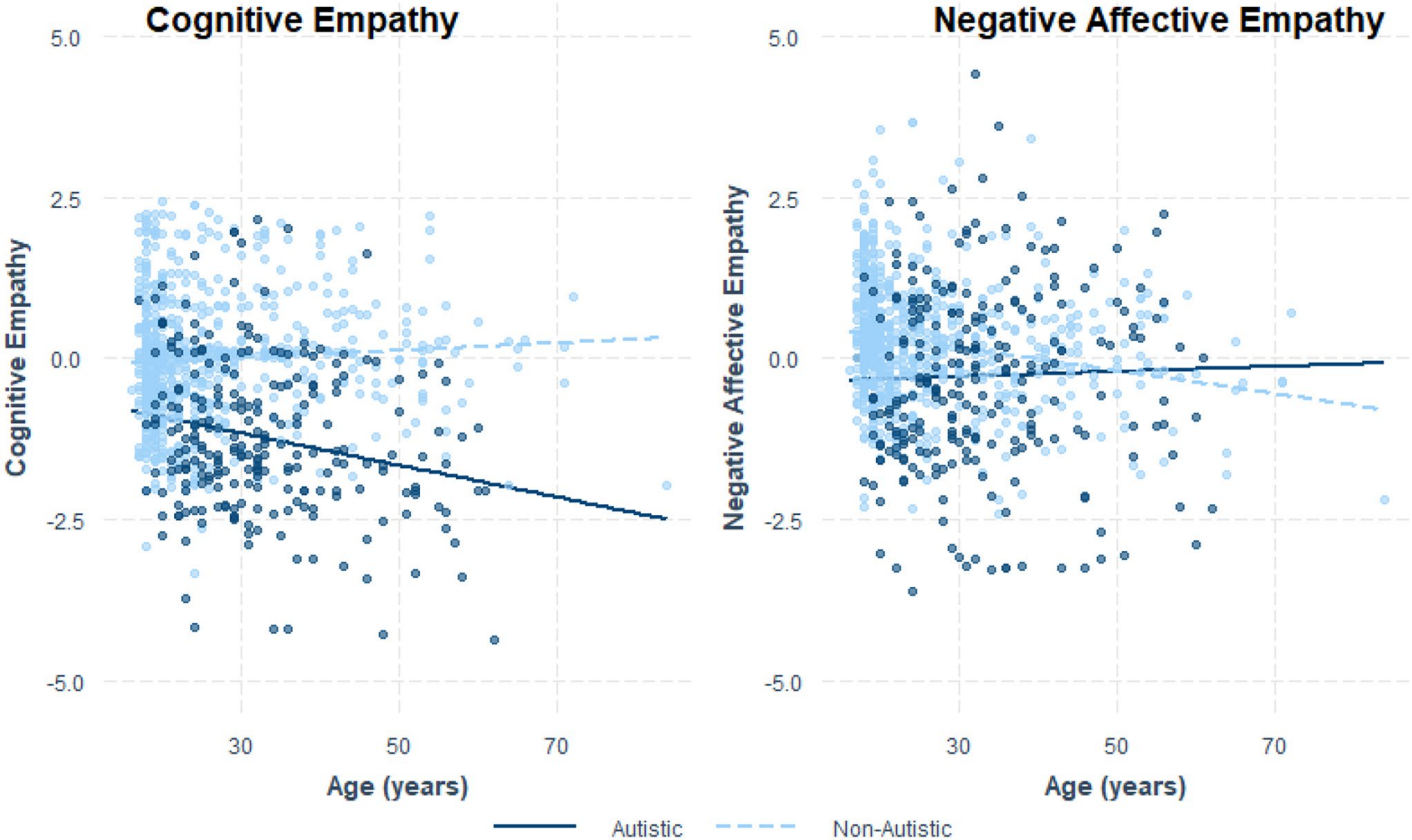
*Effects of Age in moderating the effects of autism diagnosis on cognitive empathy and negative affective empathy*

The analysis indicated that two of the main effects of autism diagnosis on empathy means were moderated by age (Table 4). Specifically, age moderated the effects of autism diagnosis on cognitive empathy and affective empathy for negative emotions (see Fig. 3). For cognitive empathy, Johnson-Neyman regions of significance (controlling for false discovery rate; see Figure S1) revealed that, for all ages within our sample, non-autistic individuals showed greater cognitive empathy than autistic individuals, but that older age accentuated the difference between the two groups. For negative affective empathy, it appeared that non-autistic individuals reported reduced negative affective empathy with age, while autistic individuals reported no differences with age (trending to improve; see Fig. 3). Indeed, Johnson-Neyman regions of significance (see Figure S2) indicated that for participants younger than 39.29 years, autistic individuals reported significantly reduced empathy compared to non-autistic individuals; in contrast, for participants older than this age, autistic and non-autistic individuals did not report significantly different negative affective empathy levels.

The analysis revealed that males and females reported similar levels of cognitive and positive affective empathy (i.e., the model did not fit the data better when allowing gender to influence these factors). However, females reported greater levels of negative affective empathy than males. Interestingly, these gender differences appeared to be the same across autistic and non-autistic individuals (i.e., the model did not fit the data better when allowing moderating effects of gender).

## Discussion

The present study investigated whether the empathy construct manifests similarly across autistic and non-autistic samples. In particular, we examined whether the PES showed validity in measuring empathy between autistic and non-autistic samples and investigated the empathy profile of autism using this measure. This PES structure showed partial measurement invariance, with no meaningful DIF effects, thus the scale is suitable for validly assessing and comparing empathy levels between autistic and non-autistic individuals. The results partially supported two hypotheses: that autistic individuals report differences in empathy tendencies compared to non-autistic individuals; and that empathic abilities in the autistic population are highly heterogeneous. Contrary to our hypothesis, the autistic sample demonstrated less distinction between affective empathy for positive and negative emotions than the non-autistic sample. Lastly, age moderated the empathic differences between autistic and non-autistic individuals, while gender did not.

### Structure of Empathy in the Autistic Population

The three-factor structure for the PES reported by Brett et al. (2023) for a general adult sample was replicated here for the autistic sample, therefore supporting a general “cognitive empathy” factor across negative and positive emotions and valence-specific “negative affective empathy” and “positive affective empathy” factors. Cognitive and affective empathy are meaningfully separable components of the empathy construct in autistic individuals, and valence appears to be a much more important consideration for affective empathy than cognitive empathy. In addition, there was evidence (via a bifactor model) that a general empathic factor may be tenable. While the general empathy factor explained much of the systematic variability in the PES items, it did not explain common variance to the degree indicating the PES may be used as a unidimensional scale. As such, researchers and clinicians should take caution when using the total score as it may be missing important information that the subscales may provide. Overall, the PES was shown to assess a coherent multidimensional empathy construct in an autistic adult sample, as previously demonstrated for a general adult sample (Brett et al., 2023; Larionow & Preece, 2023).

### PES Bias

An investigation into DIF suggested that the PES shows no meaningful measurement invariance as a function of autism diagnosis, gender, age, and the interactions between these demographic variables. As such, the present results promote the PES as the first self-report empathy measure to have robust psychometrics within and between autistic and non-autistic populations, thus now allowing researchers and clinicians to confidently assess the empathy construct in a manner that does not unduly bias scores for autistic individuals.

The current study provides a significant step forward for research on empathy in autism. However, future research will still need to be conducted to evaluate the validity of the PES further. While the current study provides the first step in providing confidence that PES scores are not unduly biased for autistic individuals, further evidence is needed to see if the PES shows convergent and discriminant validity. For instance, previous empathy research has highlighted a lack of convergent validity between self-report measures and behavioural tasks (e.g., Melchers et al., 2014; Murphy & Lilienfeld, 2019). Investigating how PES relates to behavioural tasks such as the Continuous Affective Rating and Empathic Responses task (CARER; Santiesteban et al., 2021) will be a needed future endeavour.

### Profile of Empathy

The present study used the PES to assess the empathy profile related to autism comprehensively. MNLFA allowed for a detailed assessment of mean, variance, and co-variance differences in the empathy factors between autistic and non-autistic samples. The present finding that autistic people report lower cognitive empathy compared to non-autistic people is consistent with the empathy-imbalance hypothesis of autism (Smith, 2009) and previous research (Fatima & Babu, 2023; Quinde-Zlibut et al., 2021; Song et al., 2019). However, contrary to the empathy-imbalance hypothesis of autism (Smith, 2009); Quinde-Zlibut et al., (2021) findings with an alternative measure, but in line with other previous research (e.g., Fatima & Babu, 2023; Song et al., 2019), the present results suggest that, compared to non-autistic people, autistic people report lower affective empathy for both negative and positive emotions.

Differences in method between the current study and Quinde-Zlibut et al. (2021) include participant recruitment and the empathy measures used. Quinde-Zlibut et al.’s (2021) participants completed their study in person, while the present study was conducted online. The differing results, therefore, may reflect autism subgroup differences (e.g., higher employment rates, higher education levels, and a lower proportion of individuals with intellectual disability in our online study; Rødgaard et al., 2022). Additionally, previous self-report and behavioural measures have been shown to measure somewhat different constructs of empathy (e.g., Melchers et al., 2015). Lilienfeld and Strother (2020) suggest that behavioural measures, as used in Quinde-Zlibut et al. (2021), assess the maximal performance of individuals, while self-report measures capture enduring patterns of behaviour and respondents’ perceptions of that behaviour. As such, the results reported here for the PES may be driven by (1) the added complexities of empathising outside a controlled environment and (2) how autistic individuals view their ability to empathise.

Understanding why autistic individuals report lower empathy will be especially important, as factors outside of being autistic may drive this difference. Firstly, the double-empathy problem (Milton, 2012) posits that this difference may be due to in-group-out-group biases, whereby the reported empathic differences are due to the experiential and communicative differences between autistic and non-autistic individuals rather than an empathic ‘deficit’ per se. As such, empathy measures may assess an individual’s ability to empathise with non-autistic individuals, thus advantaging non-autistic individuals. Indeed, non-autistic individuals show reduced cognitive empathy towards autistic individuals (Edey et al., 2016). The PES does not specify whom the responder is empathising with, and so the questionnaire could be interpreted with reference to their tendency to empathise with members of the general public, who are mainly non-autistic. Thus, as the double-empathy problem suggests (Milton, 2012), this will potentially advantage non-autistic individuals. Perhaps future research could adapt the PES to assess individuals’ tendency to empathise with non-autistic or autistic individuals to investigate the influence of the double-empathy problem.

Secondly, under the *alexithymia hypothesis* (Bird & Cook, 2013), empathic differences may be due to the high rate of co-occurrence of alexithymia in the autistic population (Ferguson et al., 2023; Kinnaird et al., 2020) and not to autism per se. Alexithymia is a trait characterised by difficulties identifying, describing, and focusing attention on one’s own emotions (Preece et al., 2018; Preece & Gross, 2023). These difficulties have been suggested to be a transdiagnostic precursor to empathy difficulties (Valdespino et al., 2017), as individuals who struggle to understand their own emotions may then struggle to understand the emotions of others (Goldman, 2006). Previous research suggests that differences associated with autism in cognitive empathy are, at least partly, explained by co-occurring alexithymia, although this emotional awareness difficulty may not fully explain these differences (e.g., Brett & Maybery, 2022; Shah et al., 2019).

### Empathy Heterogeneity Hypothesis of Autism

The present results motivate a new hypothesis, the *empathy heterogeneity hypothesis of autism*. To our knowledge, this is the first study to test for heterogeneity of empathic abilities in the autistic population statistically. The present results indicate that the ability to share in others’ emotions is more variable in the autistic population than in the non-autistic population, and hence reinforces the notion that autism represents individuals with a constellation of different abilities. The present results align with the notion that the autistic population is highly heterogeneous, a finding previously expressed for other psychological constructs (e.g., Masi et al., 2017; Mottron & Bzdok, 2020). Given the high variability in affective empathy ability, the difference in group means shown in the results should be interpreted cautiously. On average, autistic individuals may report less tendency to share in other’s emotions, but this is not true for all autistic individuals. Previous results investigating empathy and autism show mixed results (e.g., Fatima & Babu, 2023; Song et al., 2019). The present study highlights that this may be partly caused by the variability of empathic tendencies within the autistic population.

A multitude of reasons may cause the greater affective empathy heterogeneity that was observed. While the current paper provides one empathy profile for autistic individuals, multiple profiles may exist within an autistic population. For instance, some autistic individuals may have a “hyperarousal of the empathic system” (Elcheson et al., 2018, p. 189). Future research should extend the present findings by investigating these profiles using analyses such as a latent profile analysis (e.g., Spurk et al., 2020).

While multiple empathic profiles may exist, factors may also moderate this autistic profile, creating the observed heterogeneity, such as sensory sensitivity and emotion regulation. Autistic individuals can experience a large constellation of sensory sensitivities (e.g., Tillmann et al., 2020). Srinivasan (2019, as cited in Fletcher-Watson & Bird, 2019) noted that sensory dysfunction can make autistic individuals overly tuned to others’ emotions, causing an “emotional roller coaster ride” as they attempt to deal with this. In addition, autistic individuals experience greater difficulties with emotion regulation (Cai et al., 2018). It may be that autistic individuals struggle to regulate their emotional activation, which in turn could interfere with them experiencing others’ emotional experiences. Indeed, alexithymia and its sequential effects on emotion dysregulation increase affective empathy for negative emotions in individuals exhibiting more autistic traits (Brett et al., 2024). As such, it may be that the differences in empathy identified in the current results may be dependent on levels of sensory sensitivity and emotion regulation.

### Valence-specificity of Empathy

The autistic sample showed a stronger relationship between affective empathy for positive and negative emotions than non-autistic individuals. This result suggests that valence specificity in sharing emotions is less distinct for autistic individuals and is in line with Quinde-Zlibut et al. (2022), whose results showed less distinction in valenced affective empathy for autistic compared to non-autistic individuals. These findings may imply that as autistic individuals improve in affective empathy for positive emotions, they may also improve for negative emotions.

### The Moderating Effect of Age and Gender

In line with Song et al. (2019), the present study indicates that age moderates the difference in cognitive empathy between autistic and non-autistic individuals, increasing the difference for older individuals. Our results indicate that cognitive empathy in autistic individuals may reduce with age, while it remains largely stable in non-autistic individuals. Additionally, the present results showed that age also moderated the difference in affective empathy for negative emotions between autistic and non-autistic individuals. Autistic individuals appeared to show limited change in negative affective empathy with age while non-autistic individuals showed a reduction. Further research will be needed to clarify whether this age effect is due to the development of empathy throughout the life course or reflects cohort effects. Perhaps the increased difference in cognitive empathy may be due to the influence of the double-empathy problem (Milton, 2012), whereby as autistic and non-autistic individuals age, their experiences differ more, creating greater difficulties in communicating and empathising with each other. On the other hand, the reduced difference between autistic and non-autistic individuals’ tendency to share others’ negative emotions with age may reflect a conscious effort that autistic individuals make to maintain this empathy capability. In contrast, non-autistic individuals may be less invested in maintaining the capacity to share others’ negative emotions.

Interestingly, there were no moderating effects of gender, inconsistent with Song et al.’s (2019) meta-analytical finding that gender moderated differences in affective empathy between autistic and non-autistic individuals. As such, our results suggest that females report greater negative affective empathy than males and that this difference is the same in the autistic and non-autistic populations. Additionally, there was no evidence of a gender difference in cognitive and positive affective empathy. Song et al.’s (2019) findings were taken from previous empathy questionnaires that have not investigated measurement invariance between autistic and non-autistic individuals and the interacting effect of gender (e.g., whether a bias only occurs for autistic females). The effect that Song et al. (2019) found may be due to measurement rather than differences in a latent construct. In line with the idea that the female autism phenotype (see Hull et al., 2020) includes autistic females having greater social motivation (Hiller et al., 2014) and interest in relations (Lawson, 2019), perhaps previous empathy measures’ items also assess aspects of social interactions (e.g., “Before I do something I try to consider how my friends will react to it”).

### Limitations and Future Directions

While the current paper provides meaningful contributions to the field, some important limitations must be addressed. Firstly, the PES is a self-report questionnaire that can potentially be biased by social desirability. However, there is limited evidence to suggest autistic individuals exhibit different levels of social desirability biases than non-autistic individuals (Birmingham et al., 2015). Similarly, the self-report element excluded participants, particularly autistic participants, who did not have the cognitive or literacy skills to meet the questionnaire requirements. Nonetheless, future studies should attempt to account for social desirability (see Larson, 2019). Secondly, our study did not verify the participants’ diagnoses, although this is a standard methodology given the study design (e.g., Arnold et al., 2023a, b; Bang & Igelström, 2023; Lavi & Stokes, 2023; Yew et al., 2023).

## Conclusions

The present study is the first to test and find that the empathy construct manifests similarly across autistic and non-autistic individuals. We evaluated the psychometric properties of the PES and investigated the empathy profile in autism. While researchers can now use the PES to assess empathy, clinicians can also use the measure to assess the empathic abilities of their autistic clients, for example, to identify clients reporting high affective empathy who are at greater risk of experiencing social anxiety (Pittelkow et al., 2021) and depression (Yan et al., 2021). Moreover, the present results using the PES provide greater nuance to our understanding of empathy and autism, particularly the importance of considering the heterogeneity of autism. Overall, this is the first study, to our knowledge, to statistically test and establish that an autistic sample is more heterogeneous in empathy than a non-autistic sample. This outcome highlights the need to avoid misrepresentations by generalising mean empathic differences.

## Footnotes

1 We use identity-first language as this is often what is preferred within the autistic community (Monk et al., 2022).

2 1023 participants completed the questionnaire, however 37 were removed for self-disclosing invalid data, 28 participants were removed for completing the questionnaires in less than 2 s per item, 3 were removed due to not providing their age, autism diagnosis, or gender, and 23 were removed due to being gender diverse.

3 When including the moderating effect of autism, there was no statistically significant moderation in the relationships of cognitive and negative affective empathy (B = 0.15, *p* = .083), nor cognitive and positive affective empathy (B = 0.09, *p* = .365).
